# Eggshells as natural calcium carbonate source in combination with hyaluronan as beneficial additives for bone graft materials, an *in vitro* study

**DOI:** 10.1186/s13005-015-0070-0

**Published:** 2015-04-16

**Authors:** Jörg Neunzehn, Thomas Szuwart, Hans-Peter Wiesmann

**Affiliations:** Technische Universität Dresden, Institute of Material Science, Chair for Biomaterials, Budapester Strasse 27, D-01069 Dresden, Germany; Department of Cranio-Maxillofacial Surgery, University Hospital of Muenster, Research Group Vascular Biology of Oral Structures (VABOS), Waldeyerstr 30, Muenster, 48149 Germany

**Keywords:** Calcium carbonate, Eggshell, Hyaluronan, Osteoblast, Bone, Bone graft material

## Abstract

**Introduction:**

In bone metabolism and the formation especially in bone substitution, calcium as basic module is of high importance. Different studies have shown that the use of eggshells as a bone substitute material is a promising and inexpensive alternative. In this *in vitro* study, the effects of eggshell granulate and calcium carbonate towards primary bovine osteoblasts were investigated. Hyaluronan (HA) was used as artificial extracellular matrix (ECM) for the used cells to facilitate proliferation and differentiation and to mimic the physiological requirements given by the egg *in vivo*.

**Methods:**

Hyaluronan, eggshells, a combination of hyaluronan and eggshells and CaCO_3_ were applied to the cells as additive to the used standard medium (modified High Growth Enhancement Medium) in a concentration of 0,1 g/l. The effect of the additives in the culture medium was examined by proliferation tests, immunohistochemical staining (anti-collagen type I, anti-osteopontin, anti-osteonectin and anti-osteocalcin) and kinetic oxygen measurements.

**Results:**

Our investigations revealed that all investigated additives show beneficial effect on osteoblast activity. Cell proliferation, differentiation and the metabolic activity of the differentiated cells could be influenced positively. Especially in the case cell cultures treated with eggshells the strongest effects were detected, while for the hyaluronan compared with eggshells, a weaker increase in cell activity was observed.

**Conclusion:**

In summary, it can be stated that the investigated components come into consideration as beneficial supplements for bone graft materials especially for maxillo facial surgery application.

## Introduction

In the last years, a lot of different methods and materials were used to find a proper alternative to autologous bone to treat osseous defects. The use of autologous bone is the preferred augmentation method and is therefore furthermore the gold standard.

A range of bone graft substitutes are used as alternatives to autologous grafts. The ideal bone graft material should be biocompatible, osteoinductive, osteoconductive and should have satisfactory mechanical properties. A very good solution could be the production of a composite of biomaterials which could build up all the properties needed for complete bone regeneration.

In this study, three different substances, hyaluronan, calcium carbonate and eggshell-powder were tested concerning their impact towards osteoblasts.

The mean weight of hen’s eggshells is about 5.5 g and its mean thickness is between 280 – 400 μm. The essential part of eggshell is represented by mineral with 95.1%, proteins (3.3%) and water (1.6%) of the constituents. With 37.3% of the total weight, calcium is the main mineral component (the mostly in crystalline form existing calcium is calcium carbonate (CaCO_3_) with 93.6% followed by calcium triphosphate (0.8%) and magnesium carbonate).

According to the different structures of the egg, the protein content in the eggshell could vary. The organic matrix of the eggshell consists mainly of a protein-polysaccharide complex containing 11% polysaccharides and at least 70% proteins [1]. Hydroxyproline isn’t a component of the matrix protein [2], but in amino acid composition it is close to cartilage-protein-polysaccharides [3]. Chondroitin sulphates A and B are present in the matrix and account for approximately 35% of the total polysaccharides [1].

The calcified eggshell contains an organic matrix constituting of about 3% of the eggshells weight. Furthermore, this organic part contains proteoglycans and proteins like ovocleidin 116, ovotransferrin, ovalbumin, ovocalyxin-32, ovocleidin-17, osteopontin (OPN), and lysozyme, in which some of them are able to modify eggshell calcite crystal morphology and the rate of precipitation [4,5]. Of high interest in the context of bone metabolism is OPN. This specific protein plays a significant role in calcification by increasing osteoblast adhesion onto the matrix and binds to hydroxyapatite [4,6]. Anymore two functional roles of the eggshell proteins are the regulation of eggshell mineralization, and also the antimicrobial protection of the egg and its contents [7].

As alternative for bone substitute materials eggshells have been recently used in different *in vivo* studies [8,9]. Preliminary studies have focused on the biological behavior of this natural material and, more particularly, its biocompatibility and its ability to bond to the recipient bone [10]. No toxicity or inflammatory effects of this natural material are proven. In addition to its biocompatibility, avian eggshells are more than only a kind of calcium carbonate reserve. The organic components, with a special view to the proteins represented in the eggshell could be of special interest concerning osteointegration, cell migration, cellproliferation and other important steps of bone regeneration.

With approximately 95% of calcium carbonate hen eggshell has a similar mineral composition to coral. This coralline calcium carbonate is used for years to treat bone defects in dentistry and orthopedics in a converted form as hydroxyapatite and in its natural aragonite form. Because of its application as bone graft material in form of eggshells or coralline component, calcium carbonate is another investigated substance in this study [11,6,9,12-14].

Furthermore, in this study osteoblasts are also treated with hyaluronan. Hyaluronan (HA) is a natural occurring linear polysaccharide of the extracellular matrix of nearly all vertebrate animals and also as a kind of special “biofilm” around bacteria [15]. Because of its specific biochemical and physical properties hyaluronan is a quite interesting biomaterial. Its viscoelasticity is the reason why this material is one of the most important substances of different tissues and organ systems [16]. The special feature to build up hydrogen bonding between adjacent carboxyl and N-acetyl groups when it is incorporated into aqueous solution, allows HA to maintain conformational stiffness and to retain water. In this way it is possible, that up to six liters of water could be bound up by about one gram of HA. Its physiological functions in body are shock absorption, lubrication, space filling and protein exclusion. Either more, biochemical properties of HA contain the interaction with different proteoglycans of the extracellular matrix, modulation of inflammatory cells, and scavenge of free radicals [16,17].

The *in vivo* application of HA and in combination with other biomaterials, bone graft materials and autologous bone has also shown good results concerning the wound healing process and bone remodeling [18,19].

Based up on these data the aim of this *in vitro* study is to investigate the effect of eggshells, calcium carbonate and hyaluronan as a bone substitute material by the use of proliferation tests, histological staining and cell activity/viability monitoring. The investigated substances were applied to the cells as additive to the used standard medium in a concentration of 0,1 g/l.

## Methods and materials

### Cell culture

The needed osteoblasts were derived from periosteum of calf metacarpus. It was cut into 3–6 mm^2^ pieces and transferred into culture dishes. The osteogenic layer of the periosteum specimen were placed face downwards. Osteoprogenitor cells migrate from these tissue explants [20]. For 3 weeks the explants were cultured in High Growth Enhancement Medium (ICN Biomedicals GmbH, Eschwege, Germany) supplemented with 10% fetal calf serum, 250 μg/ml amphotericin B, 10,000 IU/ml penicillin, 10,000 μg/ml streptomycin, 200 mM L-glutamine (Biochrom KG seromed^(R)^, Berlin, Germany) and 10 mM β-glycerophosphate, at 37°C and 5% CO_2_ in humidified air. The cell culture medium was replaced once a week. Cells of the first passage were used for this study. The osteoblastic character of the cells used in this study was positively proven by immune histological staining (ALP, osteopontin, osteocalcin) during cultivation.

The cells were harvested by incubation with collagenase (Biochrom KG seromed^(R)^) and tyrode solution, collected and pelleted by centrifugation. The resuspended cells were seeded on the bottom of culture dishes with densities used for the different investigations. For the tests, the cell culture conditions were equal to those used for the periosteum outgrowth culture.

The substances investigated in this study were hyaluronan (HA), eggshells, a combination of hyaluronan and eggshells and CaCO_3_. The substances were applied to the cells as additive to the used standard medium in a concentration of 0,1 g/l. The hyaluronan specimens in this study were diluted out of Ostenil® (TRB Chemedica, Germany).

### Proliferation

For the proliferation tests the cells were seeded into cell culture dishes at a concentration of approximately 10 000 cells/cm^2^. Cultures were examined regularly by light microscopy and counted at defined positions in each cell culture dish after 1d, 2d, 3d, 6d and 7d.

### Histology and Immunohistochemistry

The differentiation of the osteoblasts was characterized by determination of the synthesis of bone matrix proteins. Cells were seeded at a concentration of 60 000 cells per cm^2^ and cultivated for 4 weeks. For immunohistochemistry, the cell culture medium was decanted. The specimens were washed three times with phosphate-buffered saline (PBS). Specific antibodies were used to detect extracellular matrix proteins by immunohistochemical staining.

Anti-collagen type I, polyclonal was obtained from BioTrend Chemikalien GmbH, Germany, the antibodies anti-osteocalcin and anti-osteonectin were obtained from Takara, Shiga, Japan and anti-osteopontin from CHEMICON International Inc., Temecula, USA. For immunohistochemical staining, the DAKO EnVisionTM + −system was applied. The stained cell cultures were controlled and analysed by light microscopy. Richardson staining was accomplished with a blue dye (Methylen blue Azur II).

### Cell activity/Kinetic oxygen measurement

To monitor and analyze the cell activity the CVC-96 Cell culture monitoring system (O2-Scan GmbH, Germany) in combination with the fluorescence reader FLx800 TBI (Bio-Tek, Germany) was used. The CVC96 system allows observing and evaluating the cells metabolic rate of oxygen. CVC96 is a special lid, provided with pins for each well of a 96-well plate dipping in the culture medium without penetrating the cells. The tops of these pins are covered with gas permeable membrane including a fluorescent dye, which shows its fluorescence depending of the O_2_ content.

Low level of the O_2_ content in the medium detects higher cell activity because the oxygen in the medium is consumed by the cells in culture [21].

The cells were seeded at a concentration of 60 000 cells per cm^2^, cultivated up to the state of confluence and supplied with the different treated media and a standard medium. The cell activity of the cultures was measured after 30, 60 and 90 hours.

### Statistics

One way analysis of variance (ANOVA) was applied for statistical analysis by the use of SigmaPlot®12 (Systat Software, Inc.). P values < 0.05 were considered significant and indicated by one*. Furthermore **indicate P < 0.01, and ***P < 0.001.

The validity and applicability of the different case groups and the appropriate values for the statistical analytic was proved by the used software Sigma Plot ®12 automatically.

## Results

General findings show, that all additives investigated in this study have a beneficial effect towards osteoblast like cells activity. The cell proliferation, cell differentiation and the metabolic activity of the differentiated cells could be influenced positively.

In particular, for the cultures treated with eggshell particles the strongest effect was detected, while for the hyaluronan compared with eggshells, a weaker increase in cell activity was observed.

### Proliferation

In the studies on cell proliferation, the cells in the reference culture with standard medium developed in the typical way for osteoblast like cells.

The influence of different medium supplements on the proliferation of bovine osteoblasts is observed and evaluated over a period of seven days.

**Figure 1:**
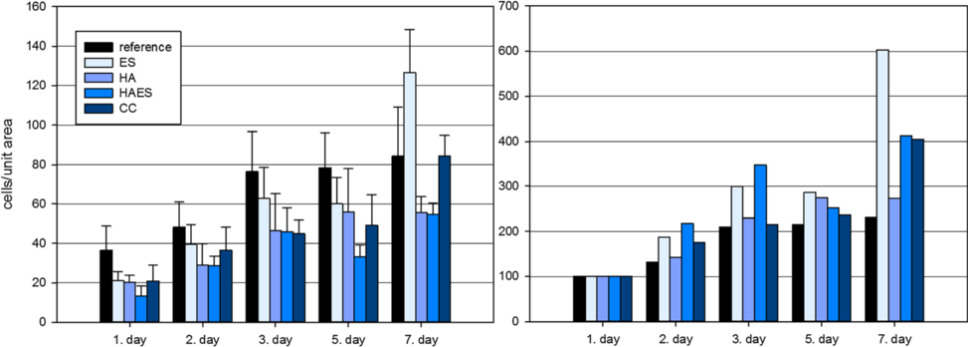
Representation of cell proliferation at an initial population of 10000 cells/cm^2^. The cell number was normalized on day 1 to 100%. (ES: eggshell powder; HA: hyaluronan; HAES: eggshell powder and hyaluronan; CC: calcium carbonate).

Figure 1 shows the proliferation profiles of the different case groups over the whole test period of seven days. The left graph represents the results of the cell counts with the corresponding standard deviations. Also in this cell proliferation study the cell numbers of the first measuring point were normalized to 100 to revise differences in the total cell amount during cell cultivation (Figure 1 right diagram).

All cell cultures treated with the active ingredients show higher cell numbers than those of the control cultures at nearly all time points (2nd, 3rd, 5th, 7th day).

While the counts of the cells treated only by the addition of hyaluronic acid (HA) increases in a negligent way, the values of the cultures treated with eggshell (ES), calcium carbonate (CC) and the combination of eggshell with hyaluronan (HAES) significantly increases for the most time points, especially after seven days. Particularly mentionable is the cell number doubling in the samples with eggshells as medium additive from fifth to seventh day.

**Figure 2:**
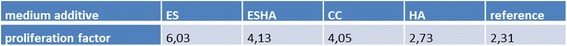
Proliferation factors of the different cell cultures (ES/eggshell, ESHA/eggshell + hyaluronan, CC/calcium carbonate, HA/hyaluronan) after seven days of cultivation.

In addition to the proliferation curves of the different treated cell cultures shown in Figure 1, the proliferation factors related to the cell populations on day 1, clarify the high cell number increases in the groups CC, HAES and PO in relation to the control cultures and the case group HA (Figure 2). This has apparently no significant proliferation-enhancing effect on the primary osteoblasts.

### Cell morphology and cell differentiation

All cultures are vital over the experimental period of 4 weeks. The cell cultures treated with eggshells and the combination eggshell/hyaluronan showed clear signs of aging. After that period the cultures were not as rich in cells and show first indication of imminent mineralization. After two weeks, all cultures are still rich in cells, but they differ occasionally significantly concerning their level of development and differentiation. Therefore, this point in time was chosen for the comparative representation. The synthesis and characterization of the degree of differentiation of the cells is shown in a Richardson overview staining and analysis of the expression pattern of the bone-specific markers collagen I, osteonectin and osteocalcin.

In all cultures, from the first week osteonectin and collagen type I are expressed clearly. From the second week, all cultures provided with a supplemented medium show distinct but partially still weak and not area-covering osteocalcin signals. Osteocalcin as an indicator for possible mineralization is particularly represented at this points.

### Richardson staining

**Figure 3:**
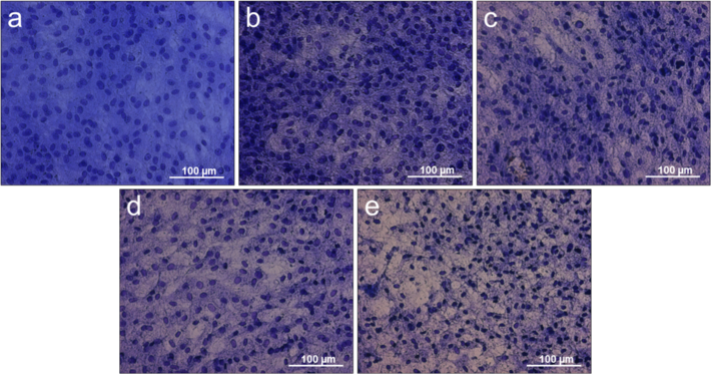
Richardson overview staining. **a)** control culture, cell cultures treated with **b)** eggshell powder (ES), **c)** calcium carbonate (CC), **d)** hyaluronan (HA), **e)** hyaluronan + eggshell powder (HAES) as cell culture medium supplement.

Figure 3 shows the results of the overview staining of a control culture and investigated case groups treated with eggshell powder, calcium carbonate, hyaluronan and hyaluronan combined with the eggshell powder as medium supplement.

In contrast to the cultures treated with the medium additives, the control group shows a very homogeneous stained cell layer with relatively even distributed nuclei, which appear very uniform in size and round-oval shape (Figure 3a). The clearest difference from the control is shown in Figure 3e, representing the HAES starved cells with a high nuclear density. The cell nuclei appear much smaller and have lost their round shape. They seem to be edgier than the nuclei of the other case groups. Intercellular open spaces have been built, streaked with a fibrillar network (Figure 3e). The cultures treated with hyaluronan (Figure 3d) and CC (Figure 3c) are very similar. Their nuclei have variations in relation of their form, but are not as small and edgy as the nuclei of the HAES cultures. In contrast to the control cultures, both treated cultures show extra- and intercellular free spaces that are filled with a fine, blue stained network structure (Figures 3c and d). The cell cultures supplied with ES represent the highest cell density with relatively closely spaced and intensively stained cell nuclei. These are surrounded by a very pronounced intercellular network structure (Figure 3b).

### Expression of type I collagen

**Figure 4:**
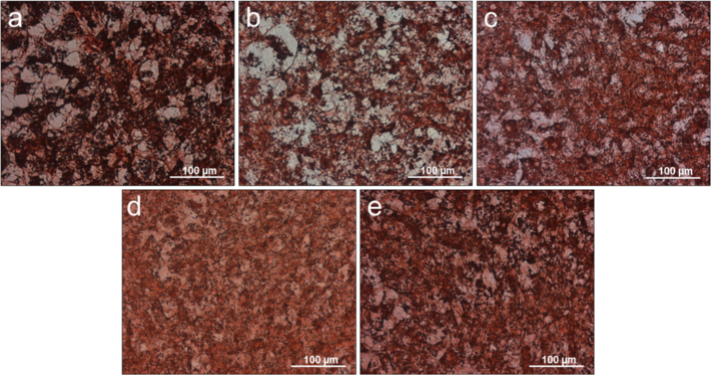
Immunohistochemistry staining with type I collagen on polystyrene. **a)** control culture, cell cultures treated with **b)** eggshell powder (ES), **c)** calcium carbonate (CC), **d)** hyaluronan (HA), **e)** hyaluronan + eggshell powder (HAES) as cell culture medium supplement.

Figure 4 illustrates the comparable collagen expression of the control cultures (Figure 4a) and the cell cultures treated with ES (Figure 4b) concerning the collagen structure formation. Intercellular unstained areas are recognizable. The collagen appears more stained in the control group (Figure 4a).

Apart from the intensity of the color, the other three case groups appear very similar. The collagen network seems to be more pronounced than the collagen structures of the control and ES cultures. The infrequent intercellular, not collagenous open spaces of the case groups HA (Figure 4d) and HAES (Figure 4e) are slightly stained in contrast to all other investigated specimens. With HAES treated cells appear to be stained more intensely than those whose medium was enriched with HA.

### Expression of osteonectin

**Figure 5:**
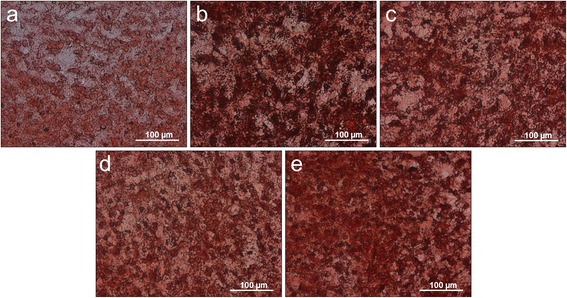
Immunohistochemistry staining with osteonectin on polystyrene. **a)** control culture, cell cultures treated with **b)** eggshell powder (ES), **c)** calcium carbonate (CC), **d)** hyaluronan (HA), **e)** hyaluronan + eggshell powder (HAES) as cell culture medium supplement.

The expression pattern of osteonectin of control, HA, CC and ES cultures are similar concerning their basic structure as shown in Figure 5. They differ, however, considerably in the intensity of staining.

The staining intensity of reference culture (Figure 5a) is less pronounced than that of HA- (Figure 5d) and CC-cultures (Figure 5c). The expression patterns of the ES sample show a much more intense staining (Figure 5b). The staining of the HAES-cultures (Figure 5e) is comparable in intensity with the HA and CC samples. However, the weaker and stronger regions of stained HAES-culture are not as clearly distinguished from each other, as in the other cultures. The different intensities merge into each other (Figure 5e).

### Expression of osteocalcin

**Figure 6:**
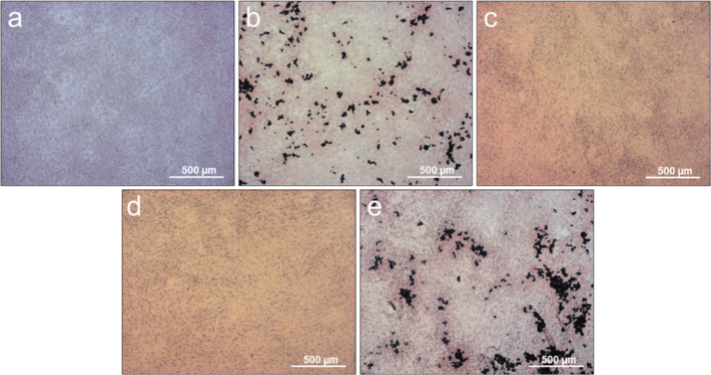
Immunohistochemistry staining with osteocalcin on polystyrene. **a)** control culture, cell cultures treated with **b)** eggshell powder (ES), **c)** calcium carbonate (CC), **d)** hyaluronan (HA), **e)** hyaluronan + eggshell powder (HAES) as cell culture medium supplement.

After the culture period of two weeks, all especially treated cultures partially represent still weak and not comprehensive osteocalcin detections. The control and the HA-cultures show the least detectable osteocalcin expression (Figure 6a and d). The osteocalcin expression of the cells treated with CC seems to be a little bit higher (Figure 6c).

The highest attested osteocalcin expression is represented in the staining of the ES (Figure 6d) and HAES (Figure 6e) starved cells. These dark stained regions can be discerned over the entire surface of the cell culture dishes. Cells treated with HAES (Figure 6e) have significantly larger stained areas, indicating an imminent mineral formation.

### Cell activity

**Figure 7:**
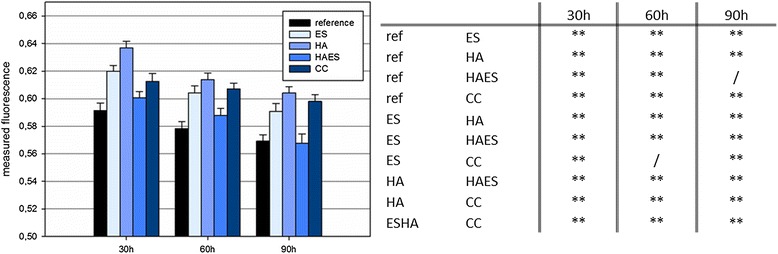
Representation of the fluorescence after 30 h, 60 h and 90 h, at an initial population of 60000 cells/cm^2^. Note: the oxygen extinguishes the fluorescence; a high fluorescence signal indicates a low oxygen level and thus a high cell activity. Results of the pairwise one-way analysis of variance (Anova) for the different case groups at the measured time points after 30, 60 und 90 hours P values < 0.05 were considered significant and indicated by one asterisk. Furthermore **indicate P < 0.01, and ***P < 0.001 (table on the right side) (ES: eggshell powder; HA: hyaluronan; HAES: eggshell powder and hyaluronan; CC: calcium carbonate, ref: reference).

The diagram in Figure 7 represents the results of the oxygen concentration in the different culture media of the investigated case groups. The oxygen uptake rates of the cell cultures demonstrate the cell activity. In particular, the cell cultures treated with eggshell powder, hyaluronan and calcium carbonate show at all three time points (30, 60 and 90 hours) higher cell activities compared to the reference cell culture.

The most significant effect on the oxygen consumption, and thus the cell viability of the osteoblasts, is given by hyaluronan (HA) as medium additive followed by the medium additives ES and CC. The oxygen consumption of the cells in the cultures with the drug combination HAES is similar to these of the reference. During the whole time of 90 h a slight decrease of cell activity can be observed in all case groups.

On the last measurement time point after 90 hours the fluorescence values of the three case groups ES, HA and CC is still above the measuring values of the control cultures after 30 hours.

The tabular presentation of the statistical analysis in Figure 7, in which the results of the cell activity measurements of the individual case groups are compared pairwise with each other, represents the high significance of the results, which highlights the accuracy of the results of all case groups.

## Discussion

The basic approach of using eggshell particles for bone regeneration is not new, and some positive effects of the material were shown in different *in vivo* studies [22,6,23,11,9,10,1,24,25].

However, the procedure carried out in the past studies is usually limited to the filling of bone defects with different sized eggshell units. The proven effectiveness of the eggshell used usually depends on the particle size and type of particle treatment in these studies.

Dupoirieux et al. were able to demonstrate that smaller particles (with a diameter of approximately 50 μm) led to a more rapid bone healing *in vivo* than bigger ones (about 150 and 300 μm in diameter). The larger ostrich eggshell particles didn’t resorb completely in the same time period [10].

The bio-inspired approach to develop a bone regeneration material in which the eggshell, as natural calcium carbonate supplier is embedded in a hyaluronan hydrogel like in this study is a new promising approach.

In this approach the hyaluronan is intended to support the active transport the released ingredients of the eggshell particles to the corresponding cells and defect edges to promote the vascularization of tissue defects as well as the wound healing *in vivo*.

Huang et al. demonstrated that hyaluronan, depending on its molecular weight, promotes a significant increase in matrix mineralization of osteoblasts [26]. One further study found that the specific application of hyaluronan normalizes the osteocalcin production in osteoarthritic osteoblasts [27]. Other authors show a promoting effect of HA on bone formation and suggest that the molecular weight and concentration of HA the differentiation behavior of the cells have a decisive influence [28,29].

Due to the large number of positive, *in vitro* confirmed properties of hyaluronan, it has also been used in combination with bone replacement materials and bone matrix for bone regeneration *in vivo.* On the field of *in vivo* studies there are no results or data found in literature were hyaluronan is combined with eggshell powder to investigate its potential concerning bone regeneration, only with eggshell in combination with carrageena gel or xanthan gum gel as support system [25]. An increase of osteoid formation was shown by the use of the different carrier materials compared towards the negative control in bone defects of rats *in vivo*. Hyaluronan was as additive in this combination was not investigated in any studies before.

The basic evaluation criteria or procedures regarding the biocompatibility and efficacy of tissue replacement and regenerative materials include cell culture experiments in which the cell activity and the response of tissue cells on the materials or substances are investigated with different methods.

Each cell activity, regardless of whether migration, proliferation or differentiation consumes oxygen. Therefore, the oxygen consumption of cells in the medium, an indicator for proliferation and differentiation is also, especially in the case of primary osteoblasts, which do not divide after formation of a confluent monolayer and start to differentiate an indicator of the cell metabolic activity. The oxygen consumption of the osteoblasts was measured by CVC96-System (O2-Scan GmbH, Germany). The analysis method and the applicability of this system was shown by Plate et al. [21].

The measurement of the cell activity that has been determined from the measured amount of oxygen in the cell culture medium clearly shows that in particular the hyaluronan treated cells show an increased cellular activity. However, the “metabolic” stimulation of the primary cells under the effect of the hyaluronan, documented in Figure 7 appears to have no significant effect on cell proliferation of the treated cells shown in Figure 1.

The cell activity measurement with a starting population of 60 000 cells per cm^2^ clearly shows for all additives used in this experiment, a significantly proven increase cell activity compared to the reference cell cultures (table in Figure 2). Due to the high number of cells, it could be assumed that the cells at the differentiation level, they stopped proliferation before this measurements. Cell cultures treated with the active medium ingredients, are metabolically active in a different way, they metabolize “more” than the cells without any special treatment.

The results of the histological- and immunohistochemical detection reactions shown in Figures 3 to 6, particularly at the end of the differentiation immediately before mineral formation (shown by the high expression of osteocalcin in Figure 6) underline these findings.

In comparison to the control cultures and with hyaluronan treated cell, the investigated samples with the eggshell-powder as medium additive show a proliferation-enhancing impact towards the osteoblasts.

The higher cellular activity of the hyaluronan treated cells compared with the eggshell and calcium carbonate cell cultures are reflected both in cell proliferation and differentiation. With a proliferation factor of 6.03 after seven days cells represent the highest increase in cell number in this experimental approach.

Cell proliferation is a very important criterion for assessing the biological response to a drug in the phase prior to the actual cell differentiation. A slightly increased proliferation rate induced by eggshell powder is known both from preliminary *in vitro* studies and literature [30]. Studies concerning the mode of action of hyaluronan in combination with eggshell particles don’t exist in literature.

The results of this study provide evidence that in the late phase of proliferation a beneficial effect of the additives towards the cells is provable. The first few days and the subsequent phase of the cell behavior in *in vitro* studies mainly differ in two aspects. In the first phase of the experiment, the cells adhere to the substrate, in this case the cell culture dish, and adapt to their environment. Conditioned by the relatively small number of resettlement the cells are mainly represented as single cells. Cell-cell contacts are barely detectable in this state. At this early stage, directly after cell passage, the cells exist in a limited way as osteoblasts. Their cell type specific differentiation takes place in the following days, after reaching a confluent cell monolayer with distinct cell-cell contacts.

Despite the shown unusual cell activity and proliferation factor of 4.13 (egg shell: 6.03; control: 2.31), the cells of the case group treated with hyaluronan and eggshell-powder appear, with respect to their phenotype after two weeks compared to the other groups, significantly changed. In the overview staining in Figure 3, the cell density of these cultures appears distinctly reduced and the original round-oval shape of the nuclei is irregular, partially edged and reduced in their size. These are indicators for a far advanced osteogene differentiation. This assumption is confirmed by the analysis of the immunohistochemical investigations concerning the staining of osteocalcin in Figure 6. Osteocalcin is expressed in a much higher way and a positive response towards this antibody is proven in a higher way than in the other case groups.

The cultures treated with HAES as culture medium additive additionally represent signs of imminent mineralization and compared to the other cultures a clearly increased osteocalcin expression. The osteocalcin enriched areas are strong and significantly bordered toward their surroundings. Slightly lower but also more dominant than in the other cultures the number of comparable areas of positive osteocalcin staining is recognizable in the cells supplied with the eggshell powder without hyaluronan.

The impact of the combination of eggshell powder with hyaluronan (HAES) concerning the cell phenotype and the immunohistochemical detection of osteocalcin underline the pronounced osteogene differentiation of the cells treated with HAES, compared with the eggshell treated cells, and confirms the differentiation-enhancing effect of hyaluronan-eggshell combination on tested osteoblasts.

The present cell culture study also shows a much higher efficiency of egg shell compared with the tested calcium carbonate particles with respect to cell proliferation and differentiation. An effect which could be caused by the eggshell specific proteins embedded in the calcit mineral layers of the shell. These eggshell specific proteins like ovocleidin-17, ovocleidin-116, ococalyxin-21, −25, −32 and −36, which are responsible for the biomineralization during the eggshell development in the hen’s uterus are still represented in the eggshell and are also released in the eggshell degradation process during the embryo genetic development of the chick [10,31-36]. The allergenic potential and the influence of the eggshell’s organic components towards immune response have to be investigated *in vivo.*

## Conclusion

The results of the current *in vitro* study demonstrate the high potential of the combination of eggshell particles and hyaluronan as basic components for bone regeneration and tissue engineering.

In this study the addition of hyaluronan to eggshell particles enhances the osteogene differentiation of the cells, shown by the immunhistochemical staining, especially the osteocalcin measurements. These results correspond with the finding of other studies, where beneficial effects concerning matrix mineralization, cell differentiation and osteocalcin regulation were shown.

## References

[1] Dupoirieux L, Pourquier D, Souyris F (1995). Powdered eggshell: a pilot study on a new bone substitute for use in maxillofacial surgery. J Craniomaxillofac Surg.

[2] Leach RM (1982). Biochemistry of the organic matrix of the eggshell. Poult Sci.

[3] Baker JR, Balch DA (1962). A study of the organic material of hen’s-egg shell. Biochem J.

[4] Pines M, Knopov V, Bar A (1995). Involvement of osteopontin in egg shell formation in the laying chicken. Matrix Biol.

[5] Panheleux M, Bain M, Fernandez MS, Morales I, Gautron J, Arias JL (1999). Organic matrix composition and ultrastructure of eggshell: a comparative study. Br Poultry Sci.

[6] Durmus E, Celik I, Aydin MF, Yildirim G, Sur E (2008). Evaluation of the biocompatibility and osteoproductive activity of ostrich eggshell powder in experimentally induced calvarial defects in rabbits. J Biomed Mater Res B Appl Biomater.

[7] Rose ML, Hincke MT (2009). Protein constituents of the eggshell: eggshell-specific matrix proteins. Cell Mol Life Sci.

[8] Park JW, Jang JH, Bae SR, An CH, Suh JY (2009). Bone formation with various bone graft substitutes in critical-sized rat calvarial defect. Clin Oral Implants Res.

[9] Dupoirieux L (1999). Ostrich eggshell as a bone substitute: a preliminary report of its biological behaviour in animals–a possibility in facial reconstructive surgery. Br J Oral Maxillofac Surg.

[10] Dupoirieux L, Pourquier D, Neves M, Teot L (2001). Resorption kinetics of eggshell: an in vivo study. J Craniofac Surg.

[11] Baliga M, Davies P, Dupoirieux L (1998). Powdered eggshell in the repair of cystic cavities of the jaw. Preliminary study. Rev Stomatol Chir Maxillofac.

[12] Dupoirieux L, Costes V, Jammet P, Souyris F (1994). Experimental study on demineralized bone matrix (DBM) and coral as bone graft substitutes in maxillofacial surgery. Int J Oral Maxillofac Surg.

[13] Schopper C, Moser D, Sabbas A, Lagogiannis G, Spassova E, Konig F (2003). The fluorohydroxyapatite (FHA) FRIOS Algipore is a suitable biomaterial for the reconstruction of severely atrophic human maxillae. Clin Oral Implants Res.

[14] Schopper C, Moser D, Wanschitz F, Lagogiannis G, Spassova E, Ewers R (1999). Histomorphologic findings on human bone samples six months after bone augmentation of the maxillary sinus with Algipore. J Long Term Eff Med Implants.

[15] Pirnazar P, Wolinsky L, Nachnani S, Haake S, Pilloni A, Bernard GW (1999). Bacteriostatic effects of hyaluronic acid. J Periodontol.

[16] Laurent TC, Fraser JRE (1992). Hyaluronan. Faseb J.

[17] Laurent UBG, Fraser JRE, Engstromlaurent A, Reed RK, Dahl LB, Laurent TC (1992). Catabolism of hyaluronan in the knee-joint of the rabbit. Matrix.

[18] Rehbein M (2008). Hyaluronsäure als Trägersubstanz für bioaktive Proteine zur Optimierung der Knochendefektheilung.

[19] Hulbert S, Morrison S, Klawitter J (1972). Tissue reaction to three ceramics of porous and non-porous structures. J Biomed Mater Res.

[20] Jones SJ, Boyde A (1977). The migration of osteoblasts. Cell Tissue Res.

[21] Plate U, Polifke T, Sommer D, Wunnenberg J, Wiesmann HP (2006). Kinetic oxygen measurements by CVC96 in L-929 cell cultures. Head Face Med.

[22] Schaafsma A, van Doormaal JJ, Muskiet FA, Hofstede GJ, Pakan I, van der Veer E (2002). Positive effects of a chicken eggshell powder-enriched vitamin-mineral supplement on femoral neck bone mineral density in healthy late post-menopausal Dutch women. Br J Nutr.

[23] Durmus E, Celik I, Ozturk A, Ozkan Y, Aydin MF (2003). Evaluation of the potential beneficial effects of ostrich eggshell combined with eggshell membranes in healing of cranial defects in rabbits. J Int Med Res.

[24] Dupoirieux L, Neves M, Pourquier D (2000). Comparison of pericranium and eggshell as space fillers used in combination with guided bone regeneration: an experimental study. J Oral Maxillofac Surg.

[25] Uraz A, Gultekin SE, Senguven B, Karaduman B, Sofuoglu IP, Pehlivan S (2013). Histologic and histomorphometric assessment of eggshell-derived bone graft substitutes on bone healing in rats. J Clin Exp Dent.

[26] Huang L, Cheng YY, Koo PL, Lee KM, Qin L, Cheng JC (2003). The effect of hyaluronan on osteoblast proliferation and differentiation in rat calvarial-derived cell cultures. J Biomed Mater Res A.

[27] Lajeunesse D, Delalandre A, Martel-Pelletier J, Pelletier JP (2003). Hyaluronic acid reverses the abnormal synthetic activity of human osteoarthritic subchondral bone osteoblasts. Bone.

[28] Pilloni A, Bernard GW (1998). The effect of hyaluronan on mouse intramembranous osteogenesis in vitro. Cell Tissue Res.

[29] Sasaki T, Watanabe C (1995). Stimulation of osteoinduction in bone wound healing by high-molecular hyaluronic acid. Bone.

[30] Neunzehn J, Wiesmann H (2014). Putamen Ovi enhances biomineral formation of osteoblasts in vitro. Int J Curr Microbiol App Sci.

[31] Gautron J, Hincke MT, Mann K, Panheleux M, Bain M, McKee MD (2001). Ovocalyxin-32, a novel chicken eggshell matrix protein. isolation, amino acid sequencing, cloning, and immunocytochemical localization. J Biol Chem.

[32] Gautron J, Hincke MT, Panheleux M, Garcia-Ruiz JM, Boldicke T, Nys Y (2001). Ovotransferrin is a matrix protein of the hen eggshell membranes and basal calcified layer. Connect Tissue Res.

[33] Gautron J, Murayama E, Vignal A, Morisson M, McKee MD, Rehault S (2007). Cloning of ovocalyxin-36, a novel chicken eggshell protein related to lipopolysaccharide-binding proteins, bactericidal permeability-increasing proteins, and plunc family proteins. J Biol Chem.

[34] Hincke MT (1995). Ovalbumin is a component of the chicken eggshell matrix. Connect Tissue Res.

[35] Hincke MT, Gautron J, Panheleux M, Garcia-Ruiz J, McKee MD, Nys Y (2000). Identification and localization of lysozyme as a component of eggshell membranes and eggshell matrix. Matrix Biol.

[36] Nys Y, Gautron J, Garcia-Ruiz JM, Hincke MT (2004). Avian eggshell mineralization: biochemical and functional characterization of matrix proteins. Comptes Rendus Palevol.

